# Kinematic effects of sensorimotor foot orthoses on the gait of patients with patellofemoral pain—a randomized controlled trial

**DOI:** 10.3389/fspor.2025.1546821

**Published:** 2025-04-30

**Authors:** Steven Simon, Jonas Dully, Oliver Ludwig, Carlo Dindorf, Eva Bartaguiz, Michael Fröhlich, Stephan Becker

**Affiliations:** Department of Sports Science, RPTU University of Kaiserslautern-Landau, Kaiserslautern, Rheinland-Pfalz, Germany

**Keywords:** sensorimotor system, insoles, chondropathia patellae, gait analysis, inertial measurement units

## Abstract

**Introduction:**

Foot orthoses (FOs) are a noninvasive and cost-effective medical treatment that positively influence biomechanical factors, such as the kinematics of the lower extremities. Nevertheless, there is a research gap regarding the influence of FOs, particularly sensorimotor foot orthoses (SMFOs), on joint kinematics of the lower extremity in gait. Therefore, this randomized controlled clinical trial addressed the impact of SMFOs on the ankle, knee, and hip joint kinematics of patients with patellofemoral pain (PFP) in comparison to that of biomechanical foot orthoses (BMFOs).

**Methods:**

A total of 20 participants (6 men; 14 women) were part of a three-month intervention with stratified random assignment to custom-made SMFO or BMFO treatment. In the pre- and post-tests, three 12-meter gait walks were assessed by inertial measurement units (IMUs) with the patients wearing no FOs, SMFOs, and BMFOs. For each joint in all three dimensions, three-way repeated-measures statistical parametric mapping (SPM) was performed using analysis of variance (ANOVA)-like statistics. *post-hoc*, the significant results were checked using *post-hoc t*-test-like SPMs.

**Results:**

Results show that SMFOs and BMFOs both significantly changed ankle and knee kinematic parameters in patients with PFP in long-term. No significant immediate effects of FOs were detected; however, there were significant interaction effects between the time of measurement and the groups. In the pre-post comparison, the SMFO-treated group showed less dorsiflexion in the initial contact and terminal stance, less knee flexion in the mid stance, terminal stance, and pre-swing, as well as a more neutral knee movement in the frontal plane. The BMFO-treated group showed slightly more knee abduction in the terminal stance, greater knee flexion at initial contact, and less hip adduction at initial contact.

**Conclusions:**

Overall, the results of this trial support the assumption that temporal adaptation processes play a vital role in the application of custom-made orthopedic FOs and highlight the long-term effects on the kinematics of the lower extremities.

## 1 Introduction

Orthopedic foot orthoses (FOs) are medical aids to help patients who have pain in the lower extremities (1), especially patients with distal ankle instability (2), high pronation in gait and running (3, 4), and knee pain (5, 6). Depending on the cause and symptoms, FOs are customized to achieve more favorable kinematic (e.g., joint angle) and kinetic (e.g., force peaks) conditions for lower extremity joint pain relief (7, 8). Further, FOs have been proven to reduce pain in different pathologies of the lower extremities (9, 10). Patellofemoral pain (PFP) syndrome is one of the most common causes of anterior knee pain in adolescents and adults (11, 12). Static and dynamic components, including axial and rotational errors of the lower extremity (13) and foot malalignment (14) might be the causative factors for PFP. Foot malalignments with excessive or insufficient pronation of the foot influence knee abduction moment in the frontal plane and ground reaction forces (15). Therefore, from a biomechanical perspective, the aim of the practice is to redirect the forces acting on the femoropatellar joint. Saxena et al. (16) concluded that FOs are an effective treatment option for relieving the clinical symptoms of PFP, particularly in young people. Lewinson et al. (17) investigated the potential of modifying the angular impulse magnitude of knee abduction through lateral and medial wedged FOs to alleviate pain in runners with patellofemoral pain (PFP) and found a clinically significant pain reduction. Gross and Foxworth (18) stated that FOs have a positive impact on patients with PFP with excessive foot pronation, lower extremity alignment, and an increased Q-angle.

In orthopedic care, a distinction is made between the two main approaches of custom-made FOs: biomechanical (BMFOs) and sensorimotor FOs (SMFOs) (10, 19, 20). BMFOs are characterized by supporting, bedding, and shell elements that are primarily intended to provide support, correction, and relief. In contrast, SMFOs primarily influence the activity of defined muscles via the corresponding elements at specific time intervals in the step cycle in a targeted manner (19, 21). Studies have measured the influence of SMFOs on muscle activity (22, 23), joint kinematics of the foot (24) and tibia, femur rotation (25) during walking, and postural parameters (26) in different samples with and without pathologies. Chondropathia patellae, associated with impaired patellofemoral kinematics (27), was listed by Greitemann et al. (21) as an indication for SMFO. The major targets of SMFOs in patients with PFP are improved motor control and muscle activation during movement (6), improved knee guidance through motion control of the hindfoot, and consequently, reduced retropatellar pressure (19). Kerkhoff et al. (6, 28) examined prefabricated BMFOs and SMFOs and their effects on the muscle activity of the lower extremities in participants with nonspecific knee pain. Their results showed that prefabricated BMFOs and custom-made SMFOs led to different activation patterns compared to shoes without FOs during a single-leg landing test. In contrast, SMFOs increased the influence of the semitendinosus and peroneus longus muscles on the gait. Ludwig et al. (22) measured the activation effect of sensorimotor foot orthoses (SMFO) on the peroneus longus muscle, which is primarily responsible for dynamic balance control and ankle joint stability. However, the biomechanical efficiency of the FOs in patients with knee pain remains unclear (22, 28). There is a lack of clinical studies that address the different mechanisms of action on lower extremity kinematics and their clinical effect on pathologies (19).

Therefore, the authors addressed the following research question: Does wearing custom-made SMFOs alter the kinematics of the ankle, knee, and hip in patients' gait in the short- (immediate) and long-term (three months) differently from custom-made BMFOs? It was hypothesized that both FOs immediately change the kinematics of the ankle, knee, and hip joints of the diseased knee side, and based on their different mechanisms of action, SMFOs differ in their long-term effects on the patient's gait.

## 2 Methods

### 2.1 Study design

**Figure 1:**
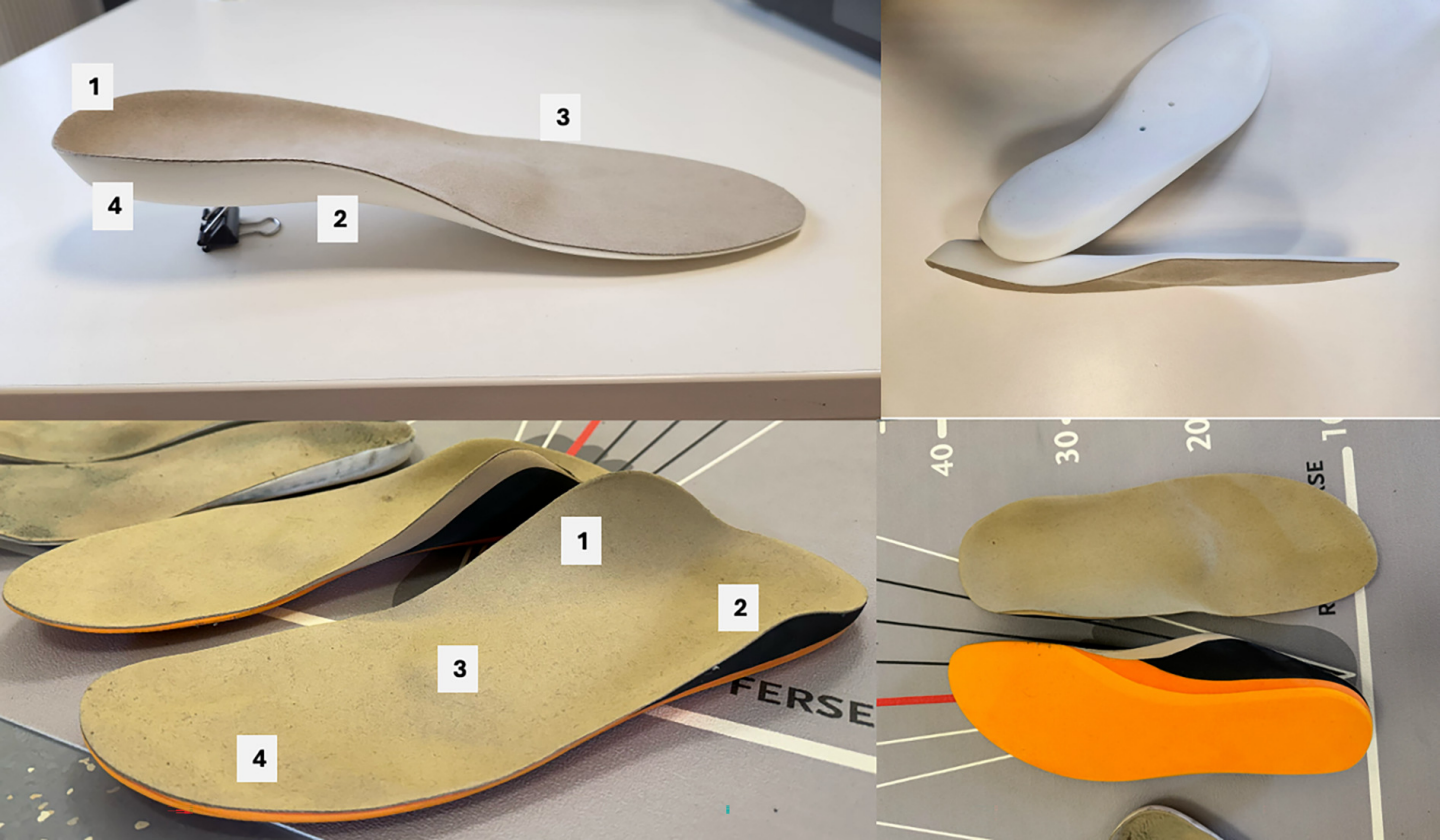
Foot orthoses (FOs) of the control group (above; BMFO, biomechanical foot orthoses) and intervention group (below; SMFO, sensorimotor foot orthoses) and. BMFO: 1 = light shell and heel pad, 2 = supination wedge, 3 = metatarsal pad, 4 = injection molded foam, 25 shore; SMFO: 1 = medial spot, 2 = lateral spot, 3 = retrocapital element, 4 = toe bar, 5 = sandwich material [ethylenvinylacetat (EVA), 35–25–35 shore].

This study represents a double-blinded, randomized-controlled clinical trial (RCT) with pre- and post-testing. The intervention period was three months. The sample was randomly assigned to an orthopedic device (SMFO or BMFO) over the intervention period after diagnostic and orthopedic anamnesis by the physician, considering the inclusion and exclusion criteria (see Chapter 2.2. and Figure 1). Placebo foot orthoses were not included in the three-month intervention due of ethical restrictions.

Scientific evaluation was carried out using the standard procedure of the physician and orthopedic shoe technician, who treat patients with corresponding orthopedic indications. The study was conducted in accordance with the local legislation and institutional requirements in accordance with the Declaration of Helsinki and approved by the Ethics Committee of the Kaiserslautern-Landau Sozialwissenschaften (Nr. 70, 16 February 2024). The study was registered in the German Clinical Register and the World Health Organization Clinical Trials Registry Platform (DRKS00035082). The participants provided their written informed consent to participate in this study.

### 2.2 Participants

A total of 26 participants were included in the baseline and follow-up gait assessments. Perceived anterior knee pain was included in another study using the same sample (29). In contrast to (29), after data processing, 20 participants were included in statistical analysis. An explanation of the dropouts in the data analysis is provided in Section 2.4.

As part of this project, another study has already been published, focusing on clinical pain development (29). A sample size of 24 participants was determined for an analysis of variance (ANOVA) to assess the interaction effect (effect size *f* = 0.25, 2 groups, 2 ToMs, *α* error probability = 0.05, correlation among repeated measures: 0.8) using G*Power 3.1 (30). The anthropometric data are shown in Table 1.

**Table 1:** Anthropometric data of sample (*n* = 20).

Groups	Descriptive statistics	Age (y)	BMI (kg/m^2^)	NI left	NI right	AI left	AI right
IG	Mean	24.40	22.34	0.16	0.16	0.20	0.20
	SD	7.65	3.78	0.02	0.03	0.07	0.06
	Max	38	31.17	0.18	0.20	0.31	0.27
	Min	15	17.26	0.12	0.13	0.09	0.09
CG	Mean	26.36	24.02	0.20	0.21	0.24	0.24
	SD	12.13	6.59	0.06	0.06	0.06	0.02
	Max	54	42.52	0.33	0.33	0.30	0.27
	Min	16	16.14	0.13	0.13	0.10	0.21

NI, navicular index (55); AI, arch index (56).

The inclusion and exclusion criteria were defined and assessed by the same physician for all participants.

**Inclusion criteria:**

• Age between 15 and 60 years
• Discomfort in the knee joint area during at least two weight-bearing activities (walking stairs, squatting, standing up) for at least three weeks: pain during these activities on most days in the last month that is ≥ 30 mm on a 100-mm Visual Analog Scale (VAS)
• Foot malalignment
• Indication (at least 1 out of 5):
  ○ Femoropatellar pain syndrome
  ○ Chondropathia patellae up to grade 3 with pathological alignment and femoral antetorsion
  ○ Runner's knee, jumper's knee
  ○ Osteochondral defects, inflammation and impingement of the Hoffa fat body,
  ○ Tendinopathies of the patellar or quadriceps tendon, patellofemoral osteoarthritis, plica syndrome
  ○ Altered Q-angle of the lower extremity ("malalignment")/recognizable rotational abnormality of ankle joints, tibia and femur during gait

**Exclusion criteria:**

• Medical history of knee joint arthroplasty or osteotomy
• Previous (surgical) treatment (<12 months) of ankle, knee, or hip joints
• Radiographic evidence of fixed bone deformity or joint erosion
• Moderate or severe concomitant tibiofemoral osteoarthritis [Kellgren and Lawrence grade 3 on anteroposterior radiograph (31)]
• Underlying neurological pathology
• Known underlying rheumatic disease with drug treatment
• Previous treatment with orthopedic foot orthoses
• Acute muscle/ligament injury (<4 weeks)

All participants were physically active. As a termination criterion during the intervention, an increase in subjectively perceived pain by two points or more during the intervention period was defined. Statistical evaluation of pain perception and development, comfort and effectiveness rating was part of another study (29). In addition, additional physiotherapeutic treatment was documented (assessed in 7 out of 20 participants), daily steps and wearing time was documented by questionnaires weekly (12 times) and the participants were asked about their dominantly pain-affected leg in case of knee pain on both sides.

### 2.3 Procedure

After anamnesis and diagnosis by a physician, the participants were instructed to attend a foot measurement appointment with an orthopedic technician. All parameters for medically indicated FO fitting were determined according to German medical standards and commissioned based on 2D foot scans and 3D foot molding. The orthopedic shoe technician was responsible for all patients. Two pairs of FOs (BMFO and SMFO) were produced for each patient and subjected to fitting and dispensing appointments. Both types of FO treatments (Figure 1) were manufactured and individually adapted to the patients' pain, foot, and knee conditions. In SMFO treatment, a medial element is positioned to apply targeted pressure along the tendon path of the tibialis posterior muscle. The lateral element is placed dorsally on the calcaneus, exerting pressure on the tendon paths of the peroneus longus and brevis muscles. The retrocapital element is positioned just behind the metatarsophalangeal joints of toes two to five. Additionally, a toe bar under the middle and distal phalanges of toes two to five provides a comfortable resting area for the distal phalanges. The design of the BMFO prioritized the height of the supination and pronation wedges, as well as the metatarsal pad, tailored to the individual's foot type (longitudinal and transversal arch posture) and clinical indication. The key advantage of these FOs lies in their soft padding, which distributes pressure preventing pressure peaks and ensuring continuous cushioning.

The orthopedic shoe technician responsible for the individual, custom-made FOs was not informed which FO was assigned to the participant during the intervention period.

First, all body dimensions (foot, leg length and width, pelvis width, and sternal height) were measured. Anamnesis, including body height, weight, activity level, and medical history, was performed. The primary research objective was to analyze various established parameters of habitual gait in the lower extremities using sensors. Therefore, Xsens inertial measurement units (IMUs) (Movella, Enschede, the Netherlands) were used. The results of Al-Amri et al. (32) suggest that the MVN BIOMECH system can be used by clinicians to quantify lower limb joint angles in clinically relevant movements. Nijmeijer et al. (2023) (33) demonstrated that the Xsens IMU system delivers highly comparable angular curves for sagittal lower-body joint kinematics during sports-specific movements, such as jump landing and change-of-direction tasks, to Vicon (Vicon Motion Systems, Ltd.).

**Figure 2:**
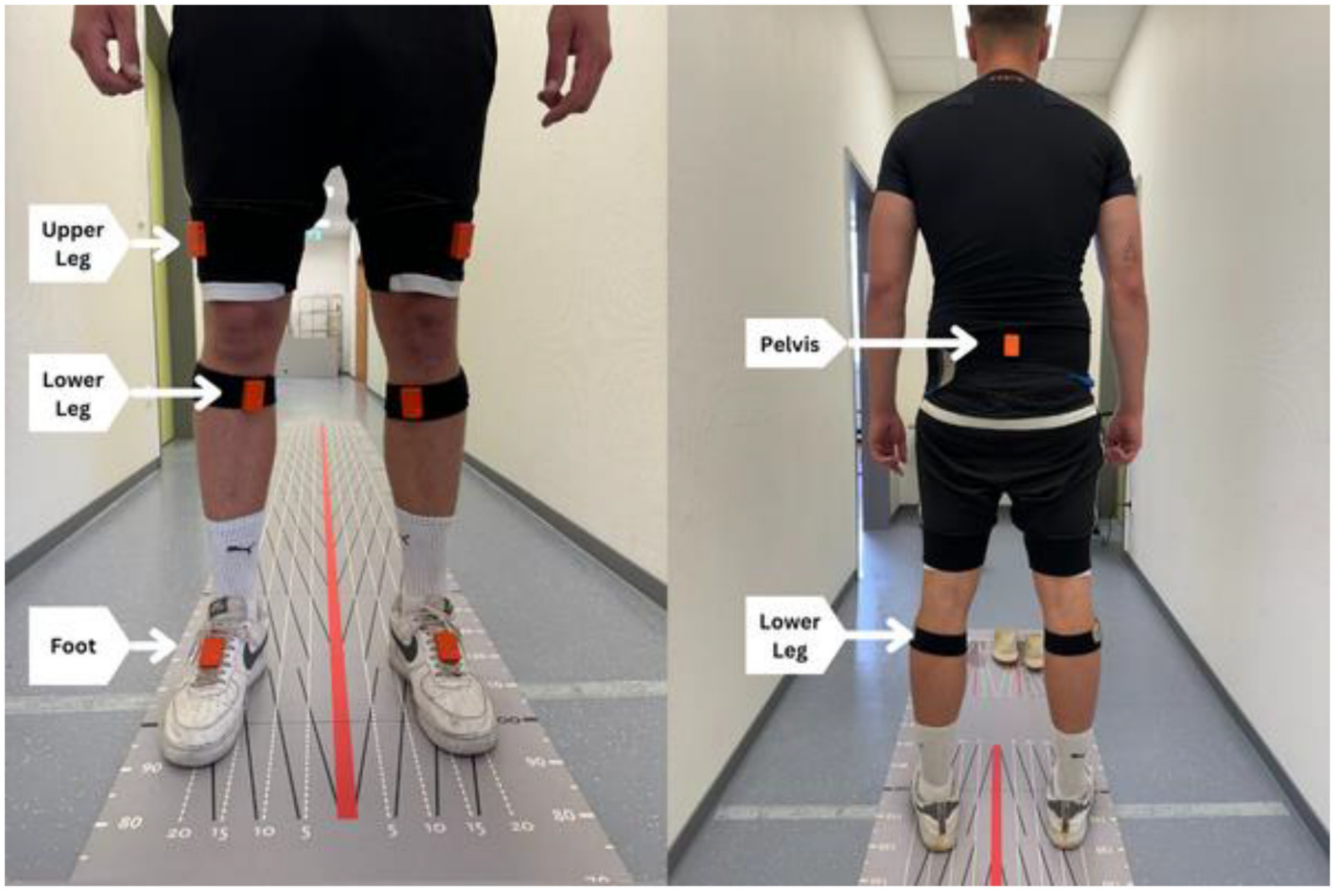
IMU-supported gait analysis with a 12-meter walking distance.

A lower-body model [60 Hz, Xsens MVN Analyze Pro 2024.2; Xsens Technologies B.V. (Enschede, The Netherlands)] represented by eight IMUs was used to measure the kinematics of the lower extremity and pelvis (see Figure 2).

**Figure 3:**
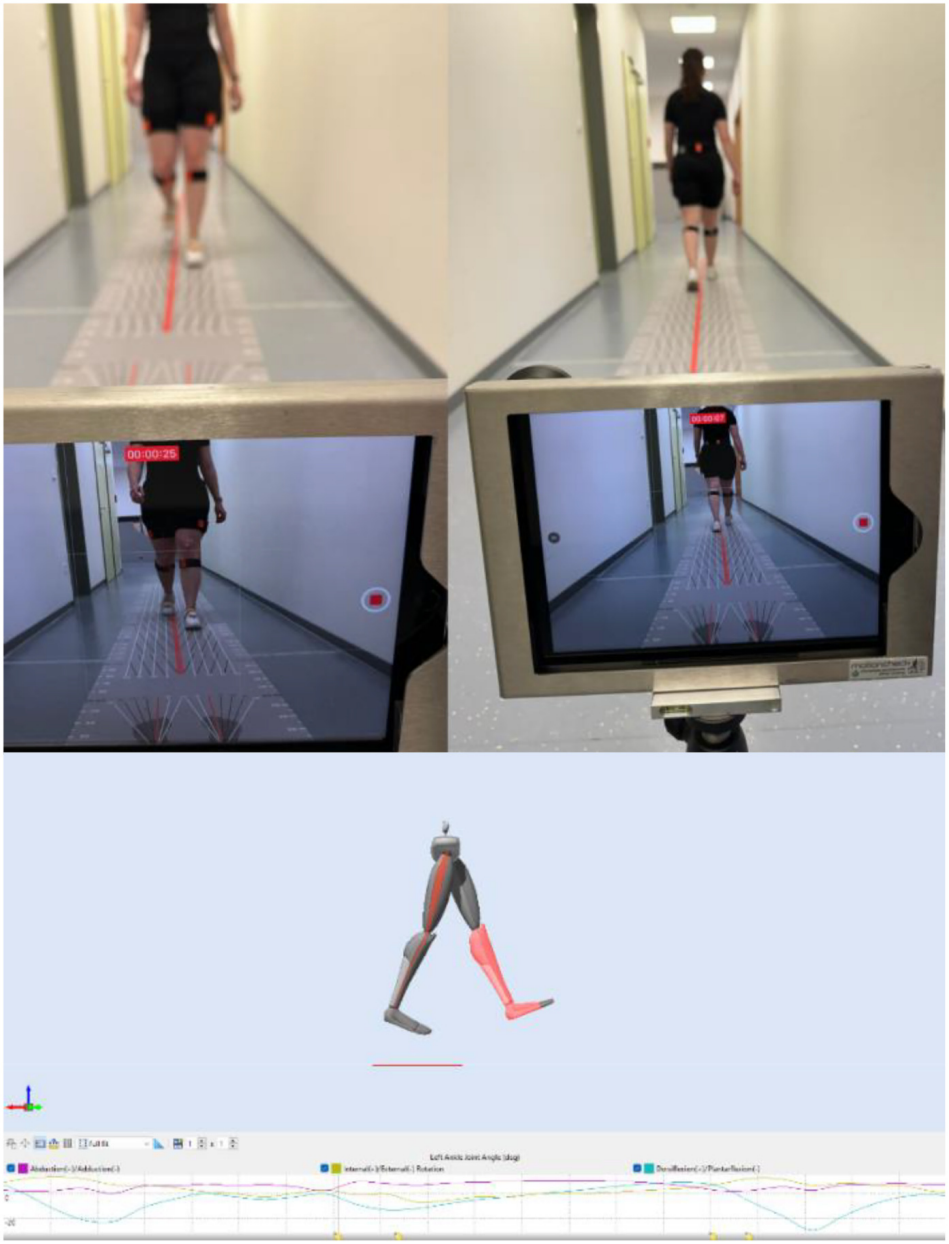
IMU placement on the patient's body (Xsens Movella, Enschede, Netherlands).

• 2x foot (each side)
• 2x lower leg (each side)
• 2x upper leg (each side)
• 1 pelvis (see Figure 3)
• 1 sternum (not shown in Figure 2)

**Figure 4:**
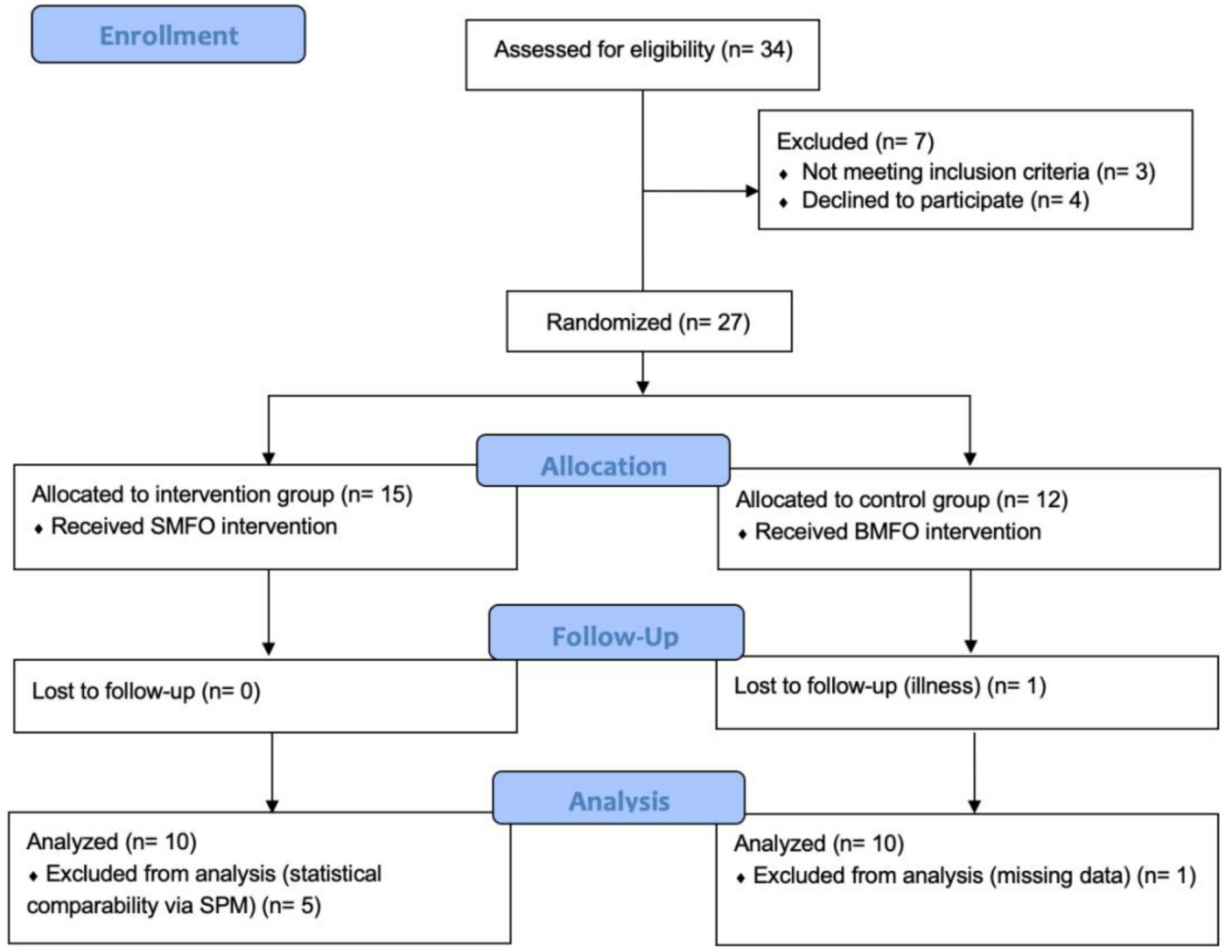
CONSORT flow diagram of this blinded randomized controlled trial (RCT). SMFO, sensorimotor foot orthoses; BMFO, biomechanical foot orthoses; SPM, statistical parametric mapping.

Each IMU comprised a three-axis accelerometer (±16 g), three-axis gyroscope (±2,000 degrees/s), and three-axis magnetometer (±1.9 Gauss) (31). These axes represent a robust and precise reference system for reconstructing three-dimensional (3D) motion. The same test leader attached the IMUs at both measurement times and fixed it to the body with a hook-and-loop fastener. They were attached to a shoe with adhesive tape. A measuring tape was used to measure the uniform distance from the knee and ankle for both measurements. The hook-and-loop fasteners protected the IMU from clothing-induced movement.

As part of the gait test, the test participant had to walk along a 12-meter gait path three times at a self-selected gait speed (Figure 3). The participant was allowed to walk the course four times in advance to become accustomed to the distance and setting. The gait speed should correspond to a patient's normal daily comfort speed. The gait distance was chosen according to the results of a systematic review by Hortobágyi et al. (34); therefore, it can be seen as a representative setting for enabling normal gait. Each participant was allowed to wear their own footwear, but the same footwear was worn during the posttest. The gait test was performed using three settings.

• 3 × 12 m with SMFOs (sensorimotor foot orthoses)
• 3 × 12 m with BMFOs (biomechanical foot orthoses)
• 3 × 12 m with NFOs (no foot orthoses)

The order of the FO in the individual shoes was randomly assigned to each participant for the pretest and structured identically in the posttest. Five gait cycles, each side cutting off the first two steps of each gait measurement, were included in the data analysis to exclude variability in the initiation and termination steps at the beginning and end of the gait cycle. After each twelve-meter distance, the patient had to stand still for three seconds to stop the measurement in the XSens Software, turn around in the starting position, and perform a new measurement. After the FO set was recorded, the test supervisor changed the FOs according to a random procedure. The participants had a five-minute break between settings.

### 2.4 Data processing and statistical analysis

After medical examination, pedographic foot measurement and motion capture were completed, and the stance phases of the gait were labeled by the test supervisor (heel strike: low point of the heel; toe off: lifting of the toe) in the Xsens MVN Analyze Pro 2024.2 software and exported in CLS files. Next, the data were merged using MATLAB (R2024b, MathWorks, Massachusetts, USA). Data acquisition resulted in three trials for each of five steps. Some participants did not have three valid trials; therefore, only two trials were used for further data processing, which were available for all subjects. This resulted in ten steps for the (dominantly) affected leg in each setting (pre- and post-training). To compare the kinematic variables (angular curves), each stance phase was normalized to 100%. The IMU-based data from the dominant PFP-affected leg were time-normalized.

For each variable, i.e., the ankle, knee, and hip angles in the sagittal and frontal planes, three-way repeated measures statistical parametric mapping (SPM) was calculated using ANOVA-like statistics with an alpha of 0.05. The two repeated measurement factors were the time of measurement (pre- and post-intervention) and the foot orthotic worn (SMFO, BMFO, NFO), whereas the non-repeated factor was the treatment group (intervention, control). Because the SPM requires a balanced design for all factors, ten participants were chosen (minimum number of participants in each group). The subjects with the most similar anthropometric characteristics between the intervention groups (SMFO and BMFO) were chosen using the Euclidean distance on z-standardized anthropometrics.

*Post hoc* for each FO, a two-way repeated-measures SPM was calculated with time and FO intervention factors. Significant results were checked using *post-hoc t*-test-like SPMs with (a) unpaired statistics for the intervention groups (SMFO and BMFO) and (b) paired statistics for the time factor. All steps were performed in MathLab (MathWorks, Natick, MA, USA) using the spm1d package (35). The results were visualized as the time-dependent group mean and standard deviation, as well as the F- or t-scores, including their critical threshold and the area under the significant results.

In addition, the anthropometric data of the groups (intervention and control) were checked with an independent Welch's *t*-test using JASP (Version 0.19.0, JASP Team, Netherlands), as few violations of sphericity and variance homogeneity were found [body mass index (BMI), navicular index].

## 3 Results

### 3.1 Consort

This study adheres to CONSORT guidelines. 34 participants were assessed for eligibility, owing to expected dropouts. 27 participants were recruited by the physician. Finally, one dropout occurred in the control group during the measurement period and six participants had to be excluded due to missing data and statistical analysis (see Figure 4 and Section 2.4).

### 3.2 Short-term effects

**Figure 5:**
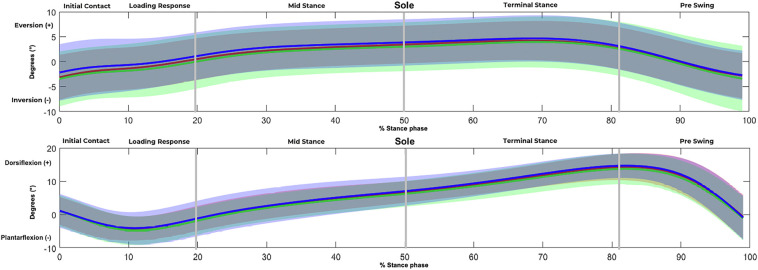
Angular curves of the ankle in the (1) frontal plane [eversion (positive), inversion (negative)] and (2) sagittal plane (dorsiflexion, plantar flexion) in the stance phases. *Y*-axis = degrees, *x*-axis = % of the stance phase. Green = SMFO, red = BMFO, blue = NFO.

Statistical analysis revealed no significant kinematic effects in both planes and all joints induced by custom-made SMFOs and BMFOs in the pretest. The descriptive data showed slightly different average angular curves in the stance phases in the frontal plane movement of the ankle. Both types of FO increased supination non-significantly in initial contact, loading response, and pre-swing and non-significantly reduced eversion in mid stance and terminal stance, whereas SMFOs showed a slightly non-significant stronger impact than BMFOs (Figure 5).

The angular curves of the knee and hip joints, as well as those in the other planes, are available in the Supplementary Material of this study. Bonferroni-corrected independent Welch's *t*-tests showed no significant differences in anthropometric data between the intervention and control groups (age: p_b_ > .99; BMI: p_b_ = .82; NI_left: p_b_ = .25; NI_right: p_b_ = .19; AI_left: p_b_ > .99; AI_right: p_b_ = .72).

### 3.3 Long-term effects

#### 3.3.1 Ankle

**Figure 6:**
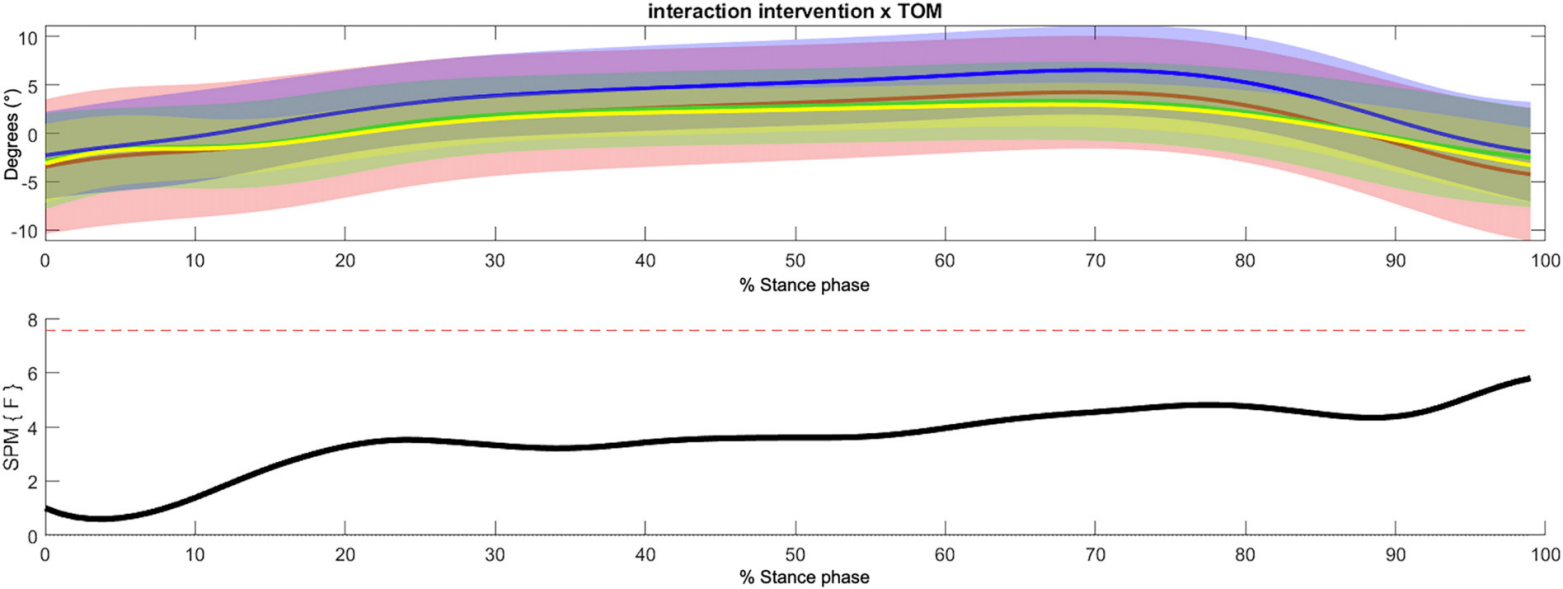
Non-significant interaction effect of intervention group and time of measurement within the 3-way repeated measures statistical parametric mapping (SPM) with averaged angular curves of the ankle joint in frontal plane. Statistical significance is present if the SPM value exceeds the threshold (red dotted line). Colors: Interaction Intervention × ToM: yellow = SMFO_pre, green = SMFO_post, red = BMFO_pre, blue = BMFO_post. Black: *F*-value.

In the frontal plane, based on the three-way SPM ANOVA-like statistics, no significant effects between groups, time of measurement, or their interaction were detected (see Figure 6).

**Figure 7:**
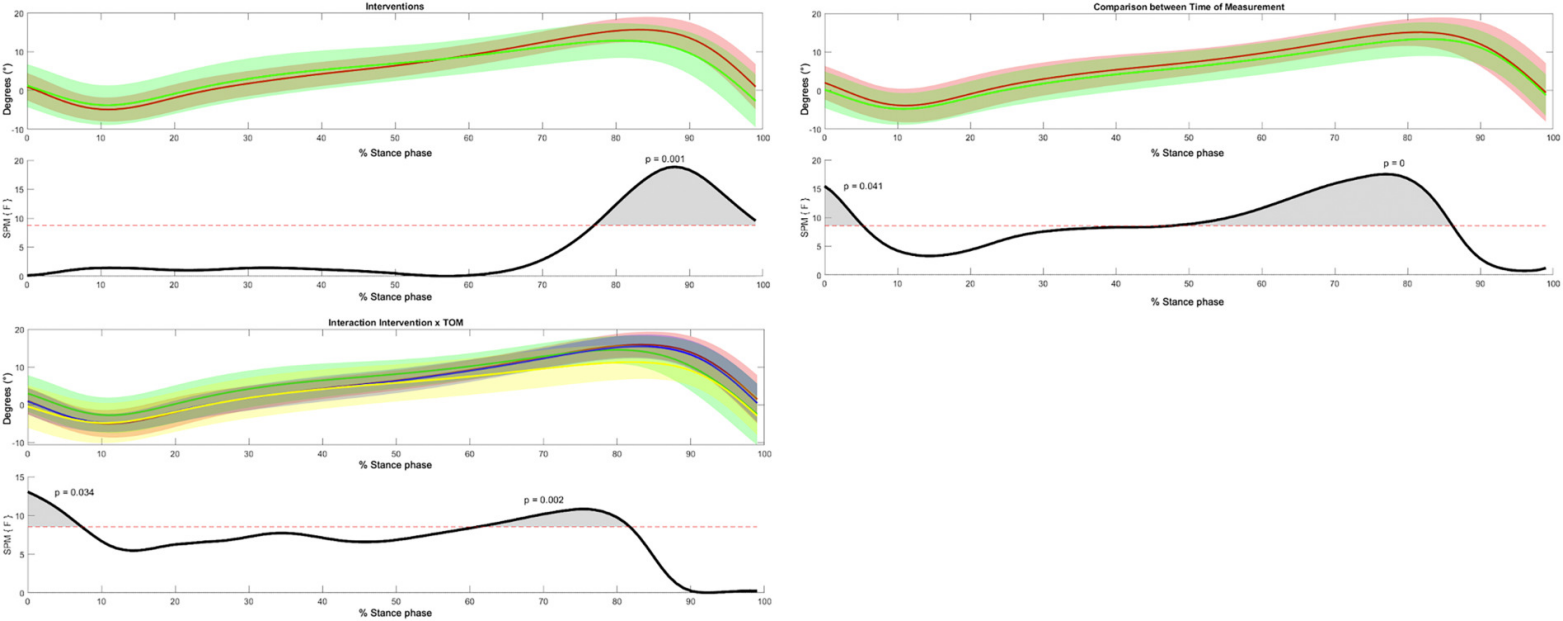
3-way repeated measures statistical parametric mapping (SPM) with averaged angular curves of the ankle joint in the sagittal plane. Statistical significance is present if the SPM value exceeds the threshold (red dotted line) represented by gray shaded areas. Colors: Interventions, green = SMFO, red = BMFO; ToM: red = pre-test, green = post-test; Interaction Intervention × ToM: yellow = SMFO_pre, green = SMFO_post, red = BMFO_pre, blue = BMFO_post. Black: *F*-value.

Regarding sagittal plane movement, three-way SPM ANOVA-like statistics showed a significant difference between foot orthoses and time of measurement, and a significant interaction effect between groups and time of measurement (Figure 7). The *post-hoc* test results are presented in Tables 2, 3. The SMFO-treated group showed reduced dorsiflexion and faster plantar flexion in the pre-swing phase compared to the BMFO-treated group. Furthermore, a significant interaction effect between the groups and the time of measurement was detected. The SMFO-treated group showed lower ankle joint dorsiflexion in the pre-swing phase. The posttest showed a significant decrease in dorsiflexion of the ankle joint in the terminal stance across all groups. The initial contact with the loading response and terminal stance showed a significant interaction effect between the groups and ToM. The SMFO-treated group exhibited the lowest dorsiflexion of the ankle joint in the posttest. In the pre-test, dorsiflexion in the SMFO group was still most pronounced at initial contact but decreased at pre-swing.

**Table 2:** Significant *post-hoc* group differences (intervention, control) in post-testing.

Comparison between intervention and control group in post-test wearing SMFO, BMFO and NFO						
Joints	Setting^a^	Initial Contact	Loading Response	Mid Stance	Terminal Stance	Pre-Swing
Ankle	NFO				Dorsiflexion: SMFO < BMFO	Plantarflexion: SMFO > BMFO
(frontal, sagittal, transversal plane)						
Knee	NFO		Abduction: SMFO < BMFO	Abduction: SMFO < BMFO	Flexion: SMFO < BMFO (end of TS)	Flexion: SMFO < BMFO
(frontal, sagittal, transversal plane)						
Hip	SMFO	Adduction: SMFO > BMFO				
(frontal, sagittal, transversal plane)						
	BMFO	Adduction: SMFO > BMFO	Adduction: SMFO > BMFO			
	NFO	Adduction: SMFO > BMFO			Adduction: SMFO < BMFO	

^a^ The column “Setting” represents the condition in which the participants wore SMFO (sensorimotor foot orthoses), BMFO (biomechanical foot orthoses) or NFO (no foot orthoses) in the gait post-test.

**Table 3:** Significant *post-hoc* time effects in intervention (SMFO) and control (BMFO) group.

Time effects		SMFO-treated group					BMFO-treated group				
(Pre vs. Post)											
Joints	Plane	Initial Contact	Loading Response	Mid Stance	Terminal Stance	Pre-Swing	Initial Contact	Loading Response	Mid Stance	Terminal Stance	Pre-Swing
Ankle	Sagittal	Dorsiflexion ↓			Dorsiflexion ↓						
Knee	Frontal	Abduction ↑	Abduction ↑	Adduction ↓						Abduction ↑	
	Sagittal			Flexion ↓	Flexion ↓	Flexion ↓	Flexion ↑				
Hip	Frontal						Adduction ↓				

SMFO-treated group = Three-month treatment with sensorimotor foot orthoses; BMFO-treated group = Three-month treatment with biomechanical foot orthoses.

#### 3.3.2 Knee

**Figure 8:**
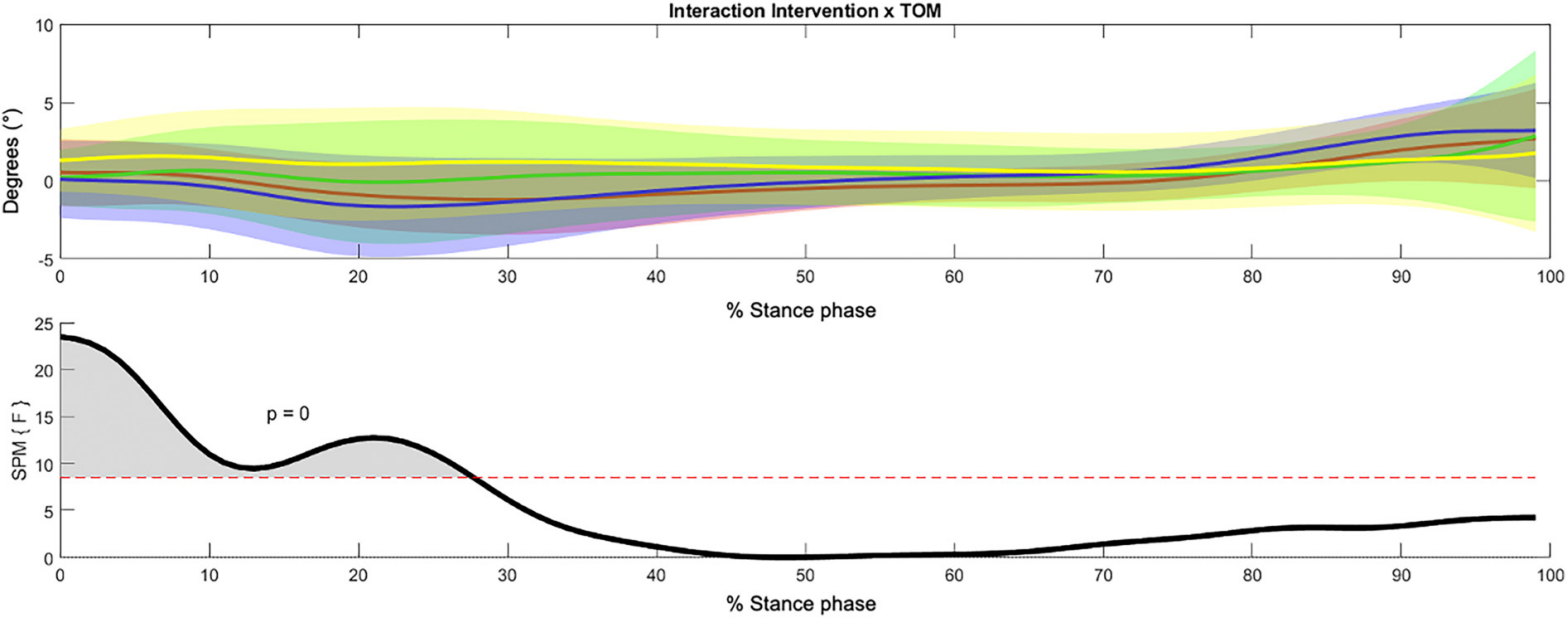
Significant interaction effect (initial contact, loading response, beginning of mid stance) of intervention group and time of measurement within the 3-way repeated measures statistical parametric mapping (SPM) with averaged angular curves of the knee joint in frontal plane. Statistical significance is present if the SPM value exceeds the threshold (red dotted line) represented by gray shaded areas. Colors: Interaction Intervention × ToM: yellow = SMFO_pre, green = SMFO_post, red = BMFO_pre, blue = BMFO_post. Black: *F*-value.

In the frontal plane, statistical analysis revealed a significant interaction effect between the intervention and time of measurement in the initial contact, loading response, and beginning of mid-stance. Particularly at the end of the loading response and beginning of mid-stance, the BMFO-treated group showed an increase in knee adduction. In contrast, the SMFO-treated group showed a normalized waveform in the range of 1–3 degrees of knee abduction (Figure 8).

**Figure 9:**
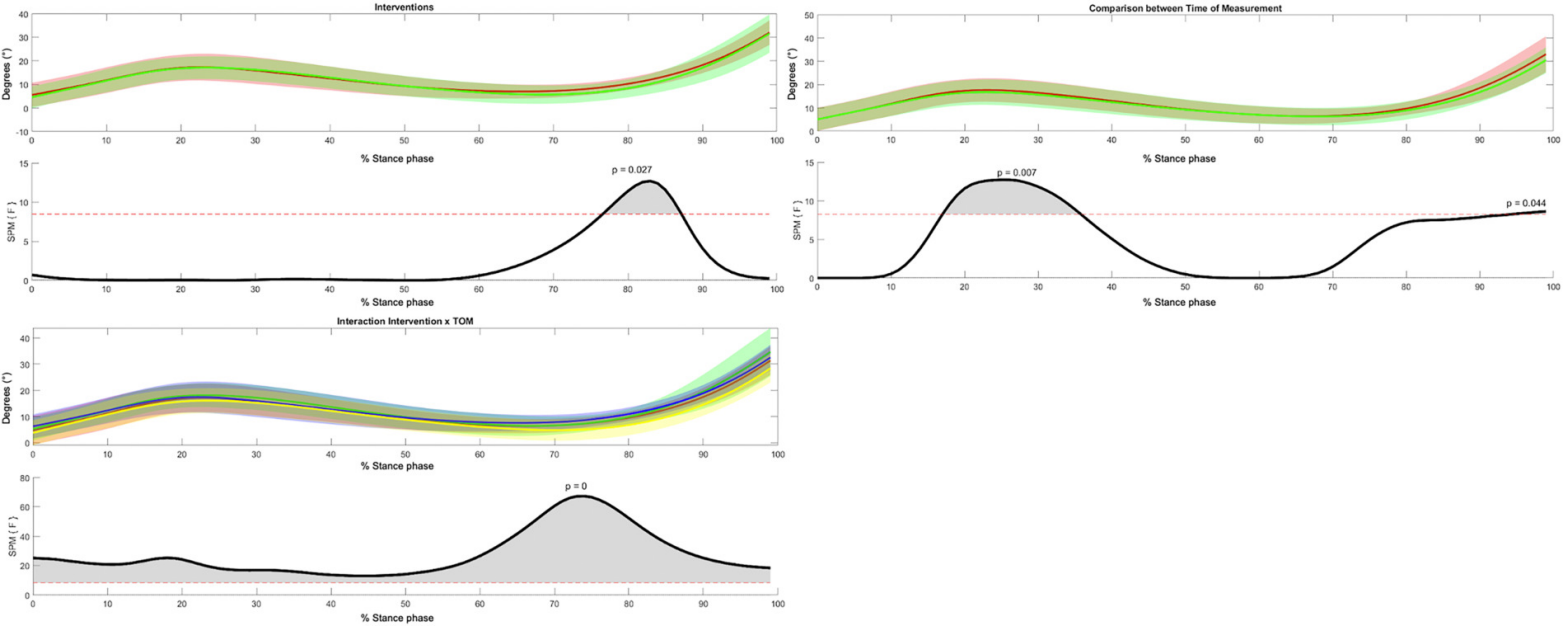
3-way repeated measures statistical parametric mapping (SPM) with average angular curves of the knee joint in the sagittal plane. Statistical significance is present if the SPM value exceeds the threshold (red dotted line) represented by gray shaded areas. Colors: Time of measurement: red = pre-test, green = post-test; interventions: green = SMFO, red = BMFO; Interaction Intervention × ToM: yellow = SMFO_pre, green = SMFO_post, red = BMFO_pre, blue = BMFO_post. Black: *F*-value.

Regarding the sagittal plane movement, significant intervention, time of measurement, and interaction effects were detected (see Figure 9). While knee flexion was reduced in the SMFO-treated group, the BMFO-treated group showed slightly stronger knee flexion. The SMFO-treated group exhibited an angular curve near the *x*-axis (0 degrees). The SMFO-treated group exhibited less knee flexion during the pre-swing period.

#### 3.3.3 Hip

In the frontal plane movement, a significant interaction effect between the intervention and ToM was detected at the initial contact, loading response, and beginning of mid-stance. The BMFO intervention group showed less hip abduction during initial contact in the post-test, whereas in the pre-test, there was slight hip adduction. *post hoc* tests, such as the SPM (Tables 2, 3), showed that the SMFO-treated group had more hip flexion in the terminal stance and early pre-swing.

## 4 Discussion

To the best of our knowledge, no study to date has examined the kinematic effects of both BMFOs and SMFOs in patients with PFP using IMUs based on an RCT study design. Results show that both SMFOs and BMFOs significantly changed ankle and knee kinematic parameters in PFP patients' gait in long-term (three months) but not in short-term.

### 4.1 Short-term effects

Although slight differences were observed in the angular curves of the assessed lower extremity joints (ankle, knee, and hip), no significant kinematic differences between the settings (SMFO, BMFO, and NFO) were detected immediately (pretest). The short-term results are in line with Laštovička et al. (36) who found no significant effects of SMFOs on the gait of asymptomatic healthy adults on the lower extremity kinematics besides hip adduction using a three-dimensional motion analysis system. Generally, FOs are intended to bring about changes in the kinematics of the lower extremities by altering the position and arches of the foot; however, there is still a lack of evidence regarding their effectiveness on kinematic changes (20, 22, 28, 37). In contrast, Klein et al. (24) observed an immediate reduction in rearfoot eversion when inserting SMFOs. Moisan et al. (38) concluded that FOs affect the biomechanics of the distal segments of the knee during most functional tasks such as step-up and step-down tests, jump landing, or stair ascent/descent. The necessity of a familiarization phase might be a possible explanation, as lower-extremity joints and their movements adapt after a certain period of wear (39, 40). The long-term results of this trial supported this hypothesis (Chapter 3). In contrast, Leung and Merseley have shown that FOs have an immediate impact on kinematics and lead to temporal improvements in gait in adults with hemiplegia (41). Nevertheless, the sample size of this study was not comparable to that of the PFP participants in this RCT. Highlighting the descriptive data of the frontal plane of the ankle, less eversion in the whole stance phase was detected when wearing BMFOs and SMFOs compared to NFOs, potentially induced by the medial support of the BMFO and the medial spot of the SMFO; however, this was not significant.

### 4.2 Long-term effects

In contrast to the short-term analysis, significant effects between groups, time of measurement, and their interactions were observed. The three-way ANOVA-like SPM revealed a significant difference between groups (intervention and control) in the sagittal angles of the ankle and knee. The BMFO-treated group showed greater dorsiflexion of the ankle in the terminal stance and pre-swing phases, while the SMFO-treated group showed reduced dorsiflexion and earlier plantar flexion of the ankle in the late stance phase. A possible explanation for that might be the proprioceptive and tactile stimulation of the SMFOs' elements on the forefoot, influencing the movement of the ankle in the phase where the most pressure is on the retro capital element and toe bar (19). Regarding the sagittal plane of the knee, significantly lower knee flexion was observed in the SMFO group than in the BMFO-treated group during the transition from the terminal stance to the beginning of the pre-swing. By reducing excessive medial or lateral knee loading, the SMFOs may shift the body's mechanics toward less reliance on knee flexion to absorb and manage forces during terminal stance. Less knee flexion during terminal stance to pre-swing could be a protective adaptation; nevertheless, both groups showed different effects because a significant interaction effect was found between the groups and the time of measurement. Generally, reduced peak knee flexion during gait is associated with PFP (11). However, studies are required to investigate this effect.

Further, time effects were detected in the sagittal angles of the ankle, hip, and knee. Both groups showed slightly less ankle dorsiflexion in the initial contact and terminal stance, less hip extension in the terminal stance, and less knee flexion in the mid-stance after the treatment period. Generally, adaptation processes may be a causative factor for these results. Hsu et al. (39) found that, after long-term use of laterally-wedged insoles, pain and physical function improved, along with a decrease in the peak knee adduction moment. The authors summarized that laterally wedged insoles provide both immediate support for walking and long-term gait adaptations that reduce stress on the knee joint in individuals with bilateral medial knee osteoarthritis. Exteroceptors and proprioceptors, as part of the sensorimotor system as well as the relevant foot and ankle muscles, may adapt movements of the lower extremity' joints according to FO treatment (19). Considering the possible biomechanical chain of the lower extremities, there appears to be a contradiction between the results of ankle, knee, and hip movements. The authors expected that reduced dorsiflexion and increased knee extension were associated with greater hip extension. In contrast, less hip extension was observed in our study, which could not be fully explained. It must be considered that measurements at multiple time points might have an influence on the results. Al-Amri et al. (32) found that for walking, the between- and within-rater reliability of discrete kinematic parameters provided by the MVN BIOMECH system ranged from fair to excellent. The ICC values for the system in the study of Al-Amri et al. were between 0.65 and 0.99, with a small standard error of measurement (SEM) of less than 3.0° for ankle and knee joints and all planes (32). These results indicate a good measurement accuracy; however, despite this high technical precision, biomechanical variability remains a crucial factor in interpreting the effects of FOs.

Significant interaction effects were observed in the sagittal and frontal angles of the knee and the frontal angle of the hip. Regarding the frontal plane of the hip joint, a slight hip abduction in the hip joint could be constantly observed with SMFO, which increased, whereas there was a slight hip adduction (in the initial contact and beginning of the loading response) with BMFO, which increased slightly. There is limited evidence highlighting the association between the peak hip adduction angle and the development of PFP in runners (11). Hoglund et al. (42) summarized that patients with PFP have altered movements during the step down test compared with asymptomatic males. Specifically, they found that PFP participants had increased hip and pelvis range of motion in the frontal and transverse planes during a step-down test in the frontal and transverse planes but reduced or nearly equal range of motion for these variables during single-leg squats. While it seems plausible that correcting foot alignment with FO makes a therapeutic contribution to the treatment of foot and Achilles tendon complaints, the extent to which FOs can contribute to knee pain due to their biomechanical or sensorimotor effects (10).

Following the *post hoc* test results of the statistical analysis, the SMFO intervention group showed reduced knee flexion at the terminal stance and pre-swing. Nevertheless, in our study the SMFO-treated group showed more neutralized frontal plane knee movement in mid-stance in pre-post comparison and less knee abduction than the BMFO-treated group in the post-test while wearing no foot orthoses. This could be explained by the different mechanisms of action of both FO concepts, whereby the biomechanical approach could guide the knee laterally via the medial wedge, whereas SMFO takes the functional chain into account by probably activating the foot supinator muscles. However, further studies are required to confirm this hypothesis.

Kinematic changes should always be interpreted in line with the therapeutic targets. This RCT included patients with PFP and multiple foot malalignments. Therefore, multifaceted kinematic effects are desirable. This RCT was additionally controlled by measuring perceived pain. The results are presented in detail in another study (29). Both interventions resulted in a significant reduction in pain between baseline and follow-up measurements, as well as over the 12-week period assessed by VAS. SMFO was perceived as more effective (Mean_Diff_ = 1.42) and slightly more comfortable (Mean_Diff_ = 0.36) than BMFO on an 11-item VAS; however, statistical analysis did not show significant differences for either parameter. Both types of FOs demonstrated a high level of comfort (BMFO: 7.91 ± 1.87; SMFO: 8.27 ± 1.10). The induced kinematic effects might help the scientific community and orthopedic aid sectors better understand the impact of different FO approaches to further improve the medical care of patients.

The participants were randomly assigned to an FO intervention group, and the anthropometric data in the group comparison showed no significant differences. Therefore, it can be assumed that age, BMI, and foot posture did not influence the statistical results and differences between the intervention groups. Physiotherapeutic treatment was documented and only conducted in 7 out of 20 participants included in this study. The statistics of (29) did not show any significant influence on the results and is not expected to influence the gait kinematics after three-month intervention. The self-selected gait speed of the participants may have been criticized for a lack of standardization. Step and stride lengths were not included in the statistical analysis of this approach; however, the supervisor inspected the participants for a comparable gait speed in all settings. Therefore, the authors assumed that gait speed might have only a minor influence on the possible differences between the FO settings. Studies such as that by Takayanagi et al. (43) showed that the average daily gait speed was lower than the average gait speed in the laboratory, but the review of Fukuchi et al. (44) highlighted that the amplitude of spatiotemporal parameters increased at faster speeds. Assuming that the participants walked relatively faster than in everyday life, this could have an amplifying effect on the kinematic findings. In the gait test, the authors did not standardize the footwear of the participants, which might have a biomechanical impact, but the same shoes had to be worn at both time of measurements.

### 4.3 Strengths and limitations

This RCT provides valuable insights into the short- and long-term kinematic effects of FOs in individuals with PFP. A major strength is the inclusion of both SMFO and BMFO, allowing for a comparative assessment of different FO approaches. The study highlights the role of adaptation processes in gait mechanics, suggesting the necessity of a familiarization phase when assessing the long-term impact of FOs. Medical examinations included the necessary diagnostic tools and examinations by the physician as proposed by Fulkerson (45). However, the diagnosis of PFP involves different symptoms and manifestations, and it is ultimately impossible to definitively prove that the functional causes of the disease are found in movements, such as altered tibiofemoral or patellofemoral mechanics (46). A major limitation, which is why studies on custom-made FOs in general and SMFOs in particular are limited, is the individuality of human anatomy and movement. Custom-made FOs must be individually adapted to the anatomical and physiological conditions of the patients. This makes comparability between participants even more difficult (19). While the current study has focused on the temporal effects in the sagittal plane of each joint, the specific contribution of each joint to gait pattern differences remains unclear. In addition, kinetic changes play a major role in PFP patients (11). Xu et al. (47) have shown that kinetic metrics play a greater than 50% role in identifying differences in gait patterns. Xu et al. (48) demonstrated that the ankle and knee joints, especially in the sagittal and transverse planes, provide crucial information for distinguishing gait features. These findings hold relevance for future studies examining FO effectiveness.

From a methodological perspective, in the biomechanical gait analysis of patients, optical Motion Capture (MoCap) systems (OMC), such as the 3D OMC from Vicon (Vicon Motion Systems Limited, Yarnton, England) or Qualisys (Qualisys AB, Göteborg, Sweden) are considered the gold standard (49). IMUs are one of the main tools used for instrumented gait analysis (50) and have been shown to have high accuracy in the sagittal plane and moderate accuracy in the frontal and transverse planes (51). Kobsar et al. (52) found that IMUs provide more accurate estimates of sagittal joint angles in the lower limbs compared to frontal or transverse angles, though it's important to note that much of this evidence remains limited. While joint kinematics generally show good-to-excellent validity and reliability in the sagittal and frontal planes, the data often come from small studies with weak statistical measures. However, the use of IMUs has the advantage that gait analyses can be carried out quickly and on site in doctors' offices, which increases the practicality and compliance of patients. In contrast to optical MoCap systems, the IMU measurement method has the disadvantage of representing the foot only as a single segment, which provides an incomplete picture of the ankle joint movement. Because FOs are expected to have a biomechanical influence on the ankle joints and this influence might also be different between the hindfoot and tibia, as well as the forefoot and hindfoot, the use of a 2-segment foot model as developed by Bauer et al. (50) might be interesting. A detailed analysis of hindfoot and midfoot motion is currently not feasible with IMU technology, as the foot and ankle are represented by just one sensor, which models them as a single rigid segment (50). To advance this field, future research must focus on improving measurement methods to generate higher-quality evidence and recommendations for these kinematic outcomes (52).

### 4.4 Future studies

In general, more RCTs are needed to investigate the kinematic changes achieved through FOs in patients with isolated foot deformities, such as pes valgus and/or planus, to compare a one-size-fits-all approach with FOs. Therefore, in case reports that do not meet the statistical standards of this study, the discrete data of individual cases with a homogeneous foot deformity pattern needs to be further investigated. Future study methodologies might be enhanced by incorporating machine learning techniques such as (53).

Additionally, larger sample sizes with balanced sexes and older age groups should be investigated in future studies. Further, more research must be conducted regarding different indications for FO treatment, as there is still no consensus in science regarding when and to what extent FOs can be used for the orthopedic treatment of different lower extremity pathologies. Musculoskeletal modeling and simulations significantly deepens the understanding of human movement and should be integrated into future efforts aiming to explore the kinetic impact of FOs on the knee joint (47, 54), particularly in patients with PFP. There is a need for further longitudinal studies investigating not only the short- but also long-term effects on the clinical and biomechanical parameters of patients.

## 5 Conclusion

In conclusion, it can be assumed that SMFOs and BMFOs both showed significant long-term effects on the ankle, knee and hip joints in PFP patients. The extent to which these changes in movement have a positive effect in the treatment of patellofemoral pain syndrome and contribute to pain reduction requires further investigation. In contrast to our hypotheses, no significant short-term effects were statistically assessed. Therefore, temporal adaption processes for custom-made FOs should be considered in clinical care. The results of this RCT further enhance the evidence base for improving the care of patients with PFP and foot malalignment using custom-made FOs. Future studies should investigate kinematic adaptions in the lower extremities induced by FOs with consistent foot malalignment and isolated pathologies

## Data Availability

The original contributions presented in the study are included in the article/Supplementary Material, further inquiries can be directed to the corresponding authors.
