# Supplementary material for: Kinematic effects of sensorimotor foot orthoses on the gait of patients with patellofemoral pain—a randomized controlled trial
Source: Front Sports Act Living. 2025 Apr 30;7:1546821. doi: 10.3389/fspor.2025.1546821 (PMC12075392; doi:10.3389/fspor.2025.1546821)
Supplement: Supplementary file 1 [file Presentation1.pptx]

## Slide 1
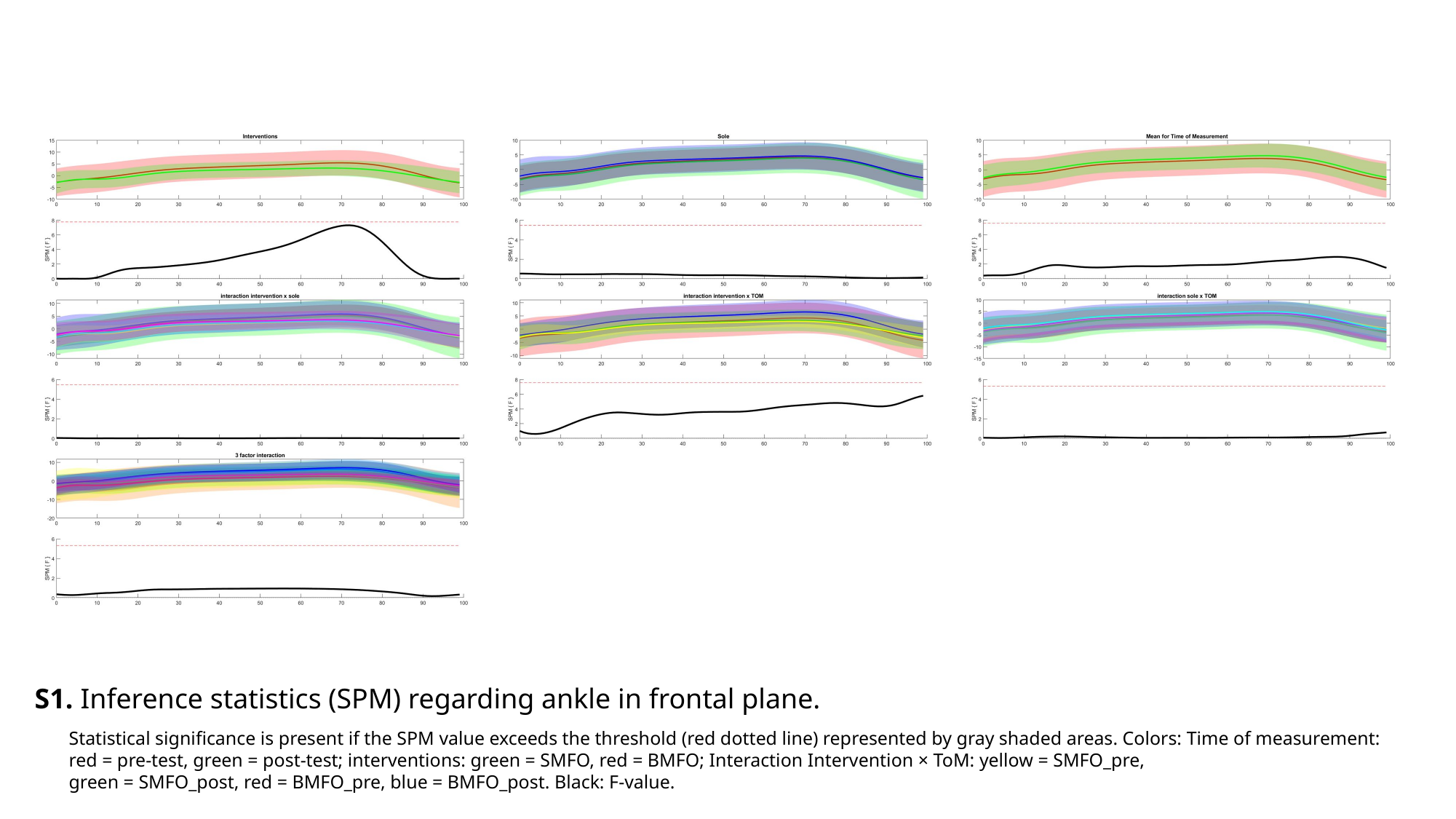

S1. Inference statistics (SPM) regarding ankle in frontal plane.
Statistical significance is present if the SPM value exceeds the threshold (red dotted line) represented by gray shaded areas. Colors: Time of measurement:
red = pre-test, green = post-test; interventions: green = SMFO, red = BMFO; Interaction Intervention × ToM: yellow = SMFO_pre,
green = SMFO_post, red = BMFO_pre, blue = BMFO_post. Black: F-value.

## Slide 2
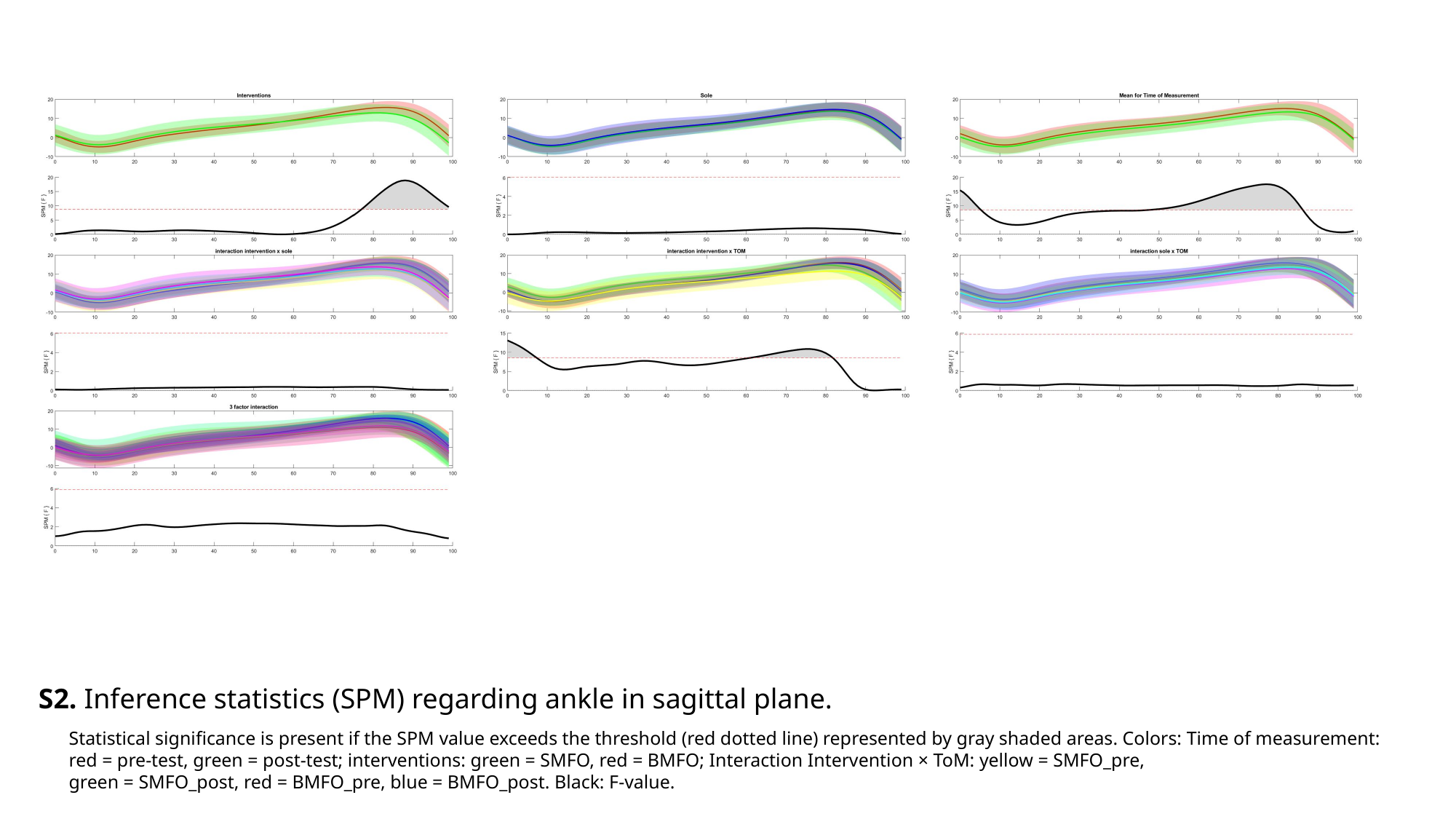

S2. Inference statistics (SPM) regarding ankle in sagittal plane.
Statistical significance is present if the SPM value exceeds the threshold (red dotted line) represented by gray shaded areas. Colors: Time of measurement:
red = pre-test, green = post-test; interventions: green = SMFO, red = BMFO; Interaction Intervention × ToM: yellow = SMFO_pre,
green = SMFO_post, red = BMFO_pre, blue = BMFO_post. Black: F-value.

## Slide 3
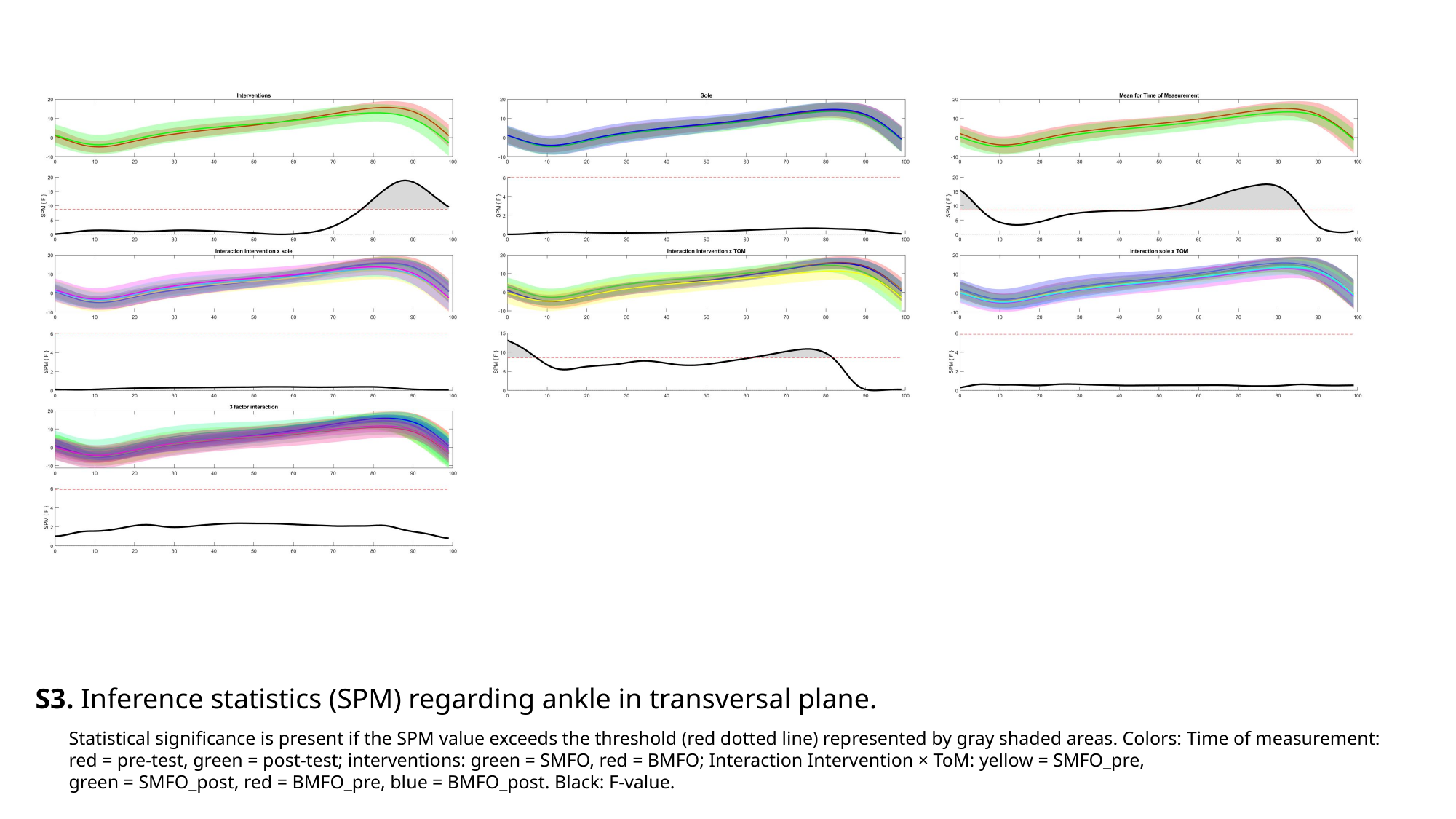

S3. Inference statistics (SPM) regarding ankle in transversal plane.
Statistical significance is present if the SPM value exceeds the threshold (red dotted line) represented by gray shaded areas. Colors: Time of measurement:
red = pre-test, green = post-test; interventions: green = SMFO, red = BMFO; Interaction Intervention × ToM: yellow = SMFO_pre,
green = SMFO_post, red = BMFO_pre, blue = BMFO_post. Black: F-value.

## Slide 4
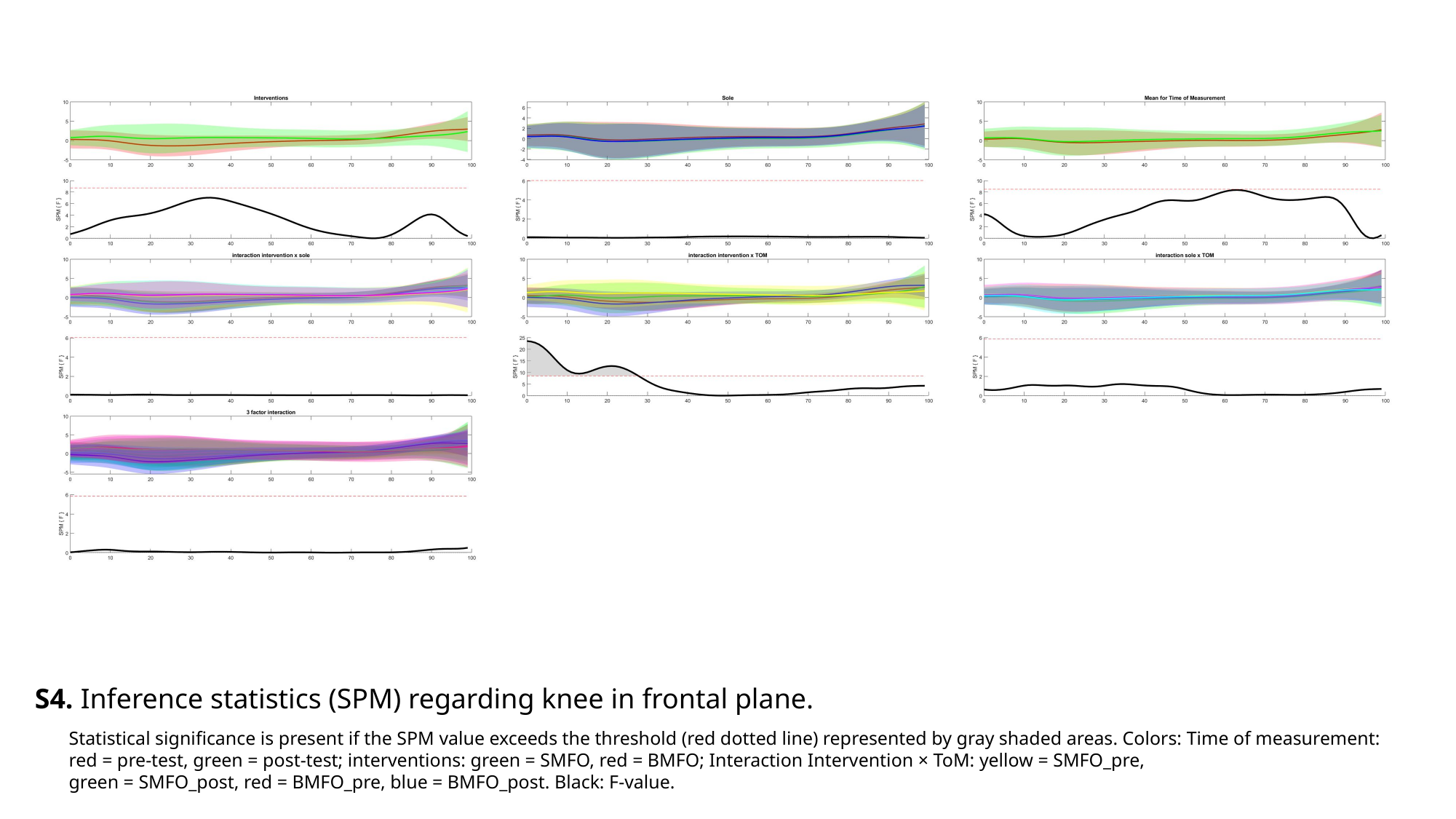

S4. Inference statistics (SPM) regarding knee in frontal plane.
Statistical significance is present if the SPM value exceeds the threshold (red dotted line) represented by gray shaded areas. Colors: Time of measurement:
red = pre-test, green = post-test; interventions: green = SMFO, red = BMFO; Interaction Intervention × ToM: yellow = SMFO_pre,
green = SMFO_post, red = BMFO_pre, blue = BMFO_post. Black: F-value.

## Slide 5
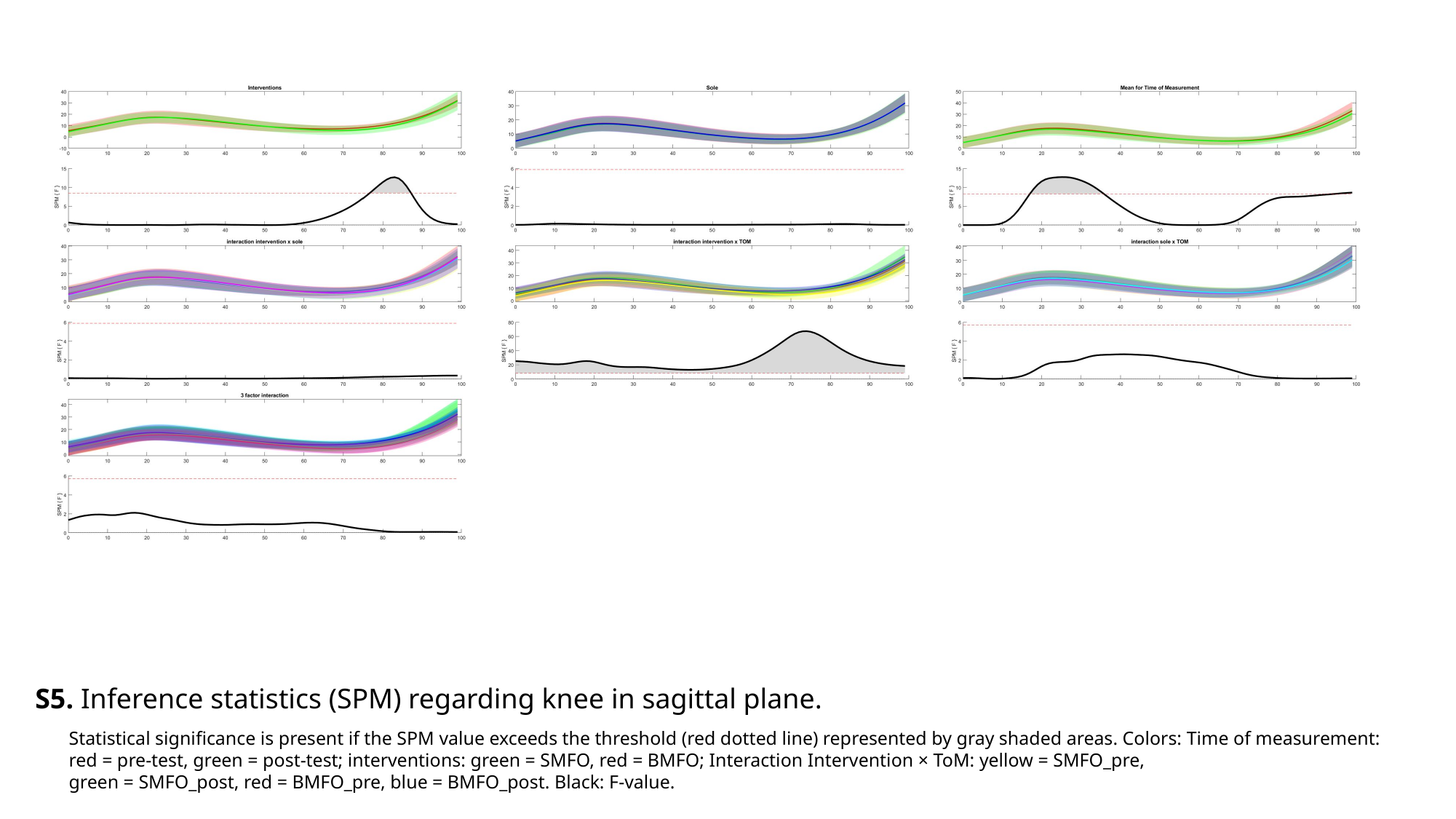

S5. Inference statistics (SPM) regarding knee in sagittal plane.
Statistical significance is present if the SPM value exceeds the threshold (red dotted line) represented by gray shaded areas. Colors: Time of measurement:
red = pre-test, green = post-test; interventions: green = SMFO, red = BMFO; Interaction Intervention × ToM: yellow = SMFO_pre,
green = SMFO_post, red = BMFO_pre, blue = BMFO_post. Black: F-value.

## Slide 6
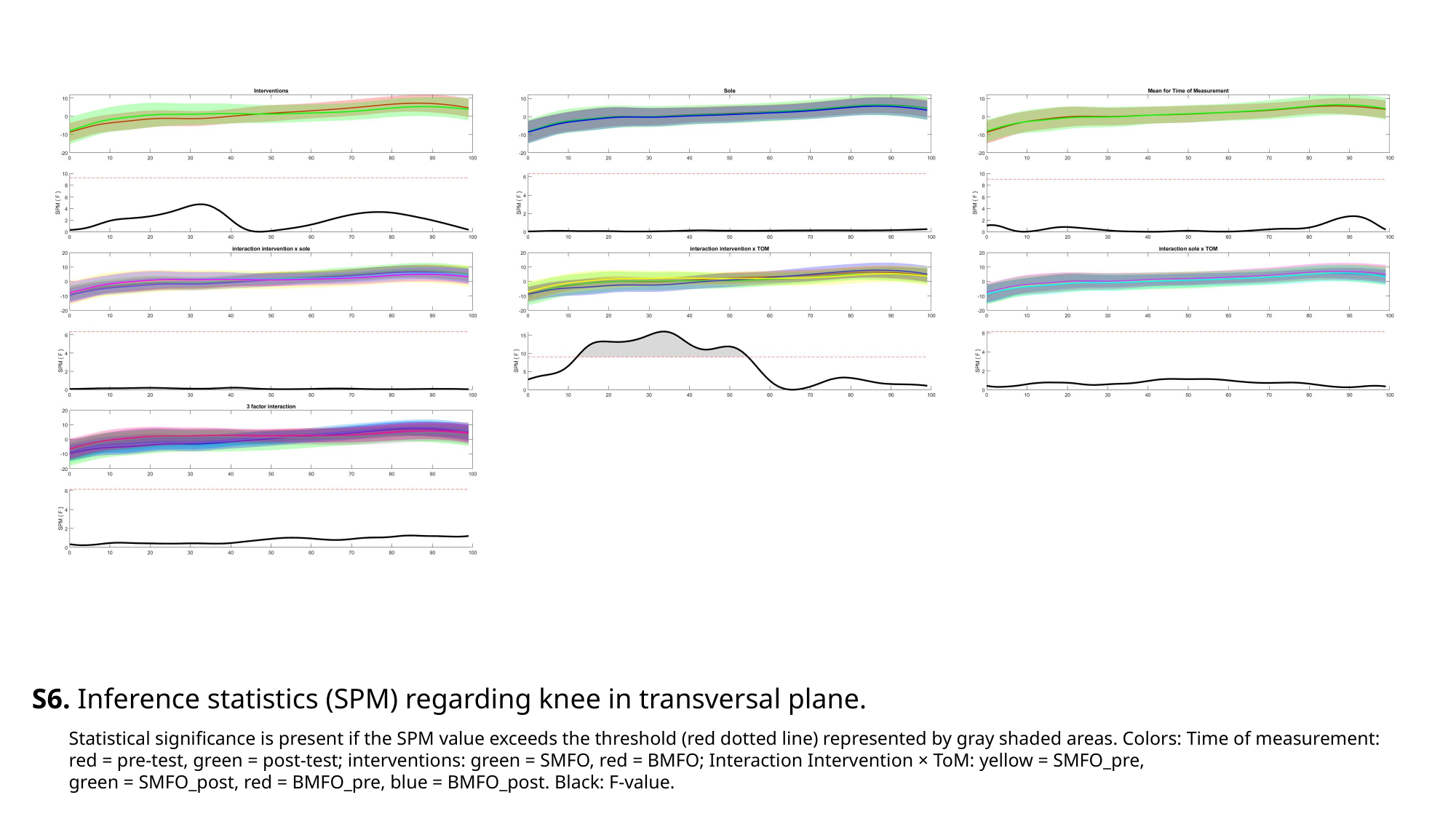

S6. Inference statistics (SPM) regarding knee in transversal plane.
Statistical significance is present if the SPM value exceeds the threshold (red dotted line) represented by gray shaded areas. Colors: Time of measurement:
red = pre-test, green = post-test; interventions: green = SMFO, red = BMFO; Interaction Intervention × ToM: yellow = SMFO_pre,
green = SMFO_post, red = BMFO_pre, blue = BMFO_post. Black: F-value.

## Slide 7
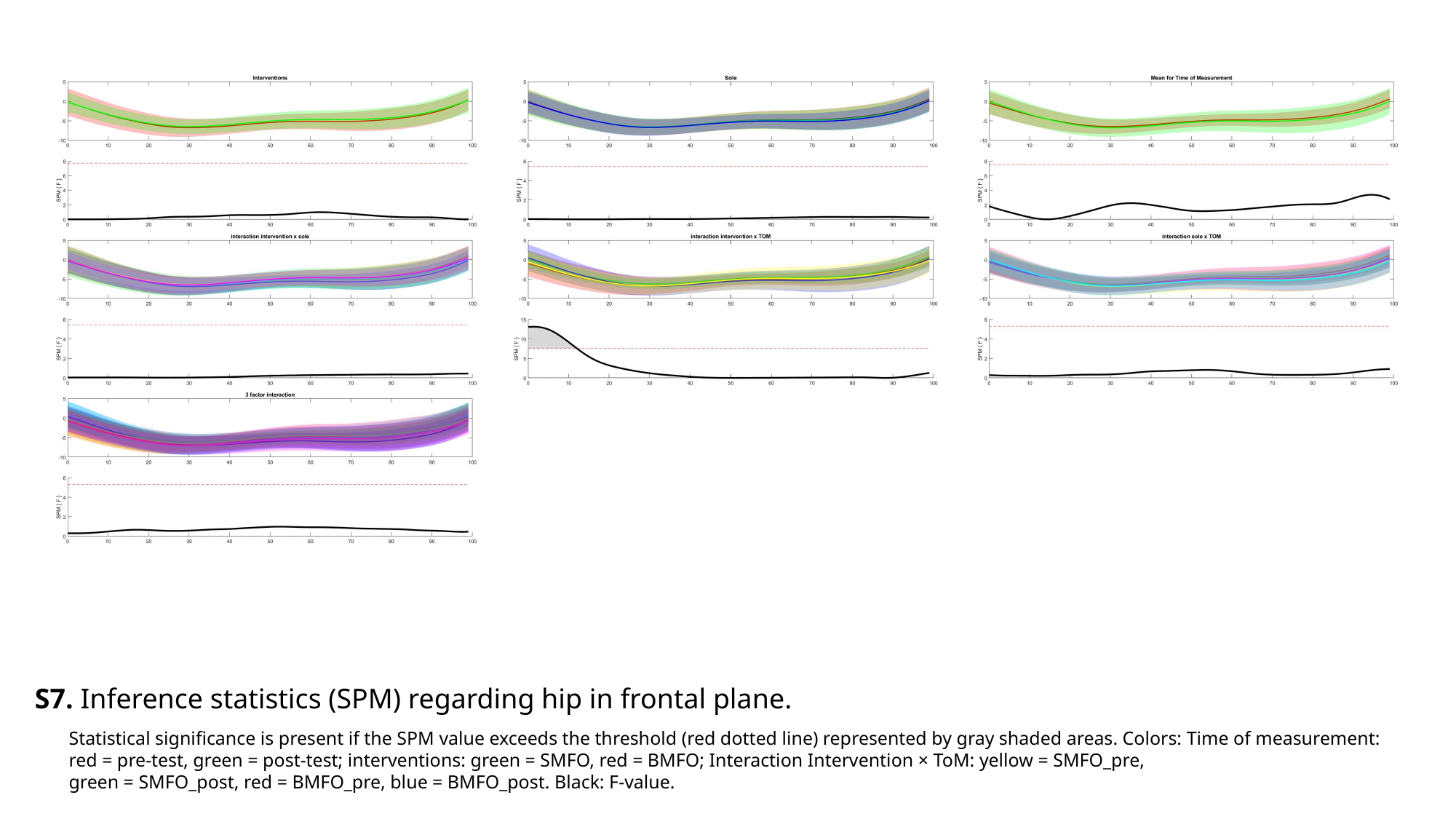

S7. Inference statistics (SPM) regarding hip in frontal plane.
Statistical significance is present if the SPM value exceeds the threshold (red dotted line) represented by gray shaded areas. Colors: Time of measurement:
red = pre-test, green = post-test; interventions: green = SMFO, red = BMFO; Interaction Intervention × ToM: yellow = SMFO_pre,
green = SMFO_post, red = BMFO_pre, blue = BMFO_post. Black: F-value.

## Slide 8
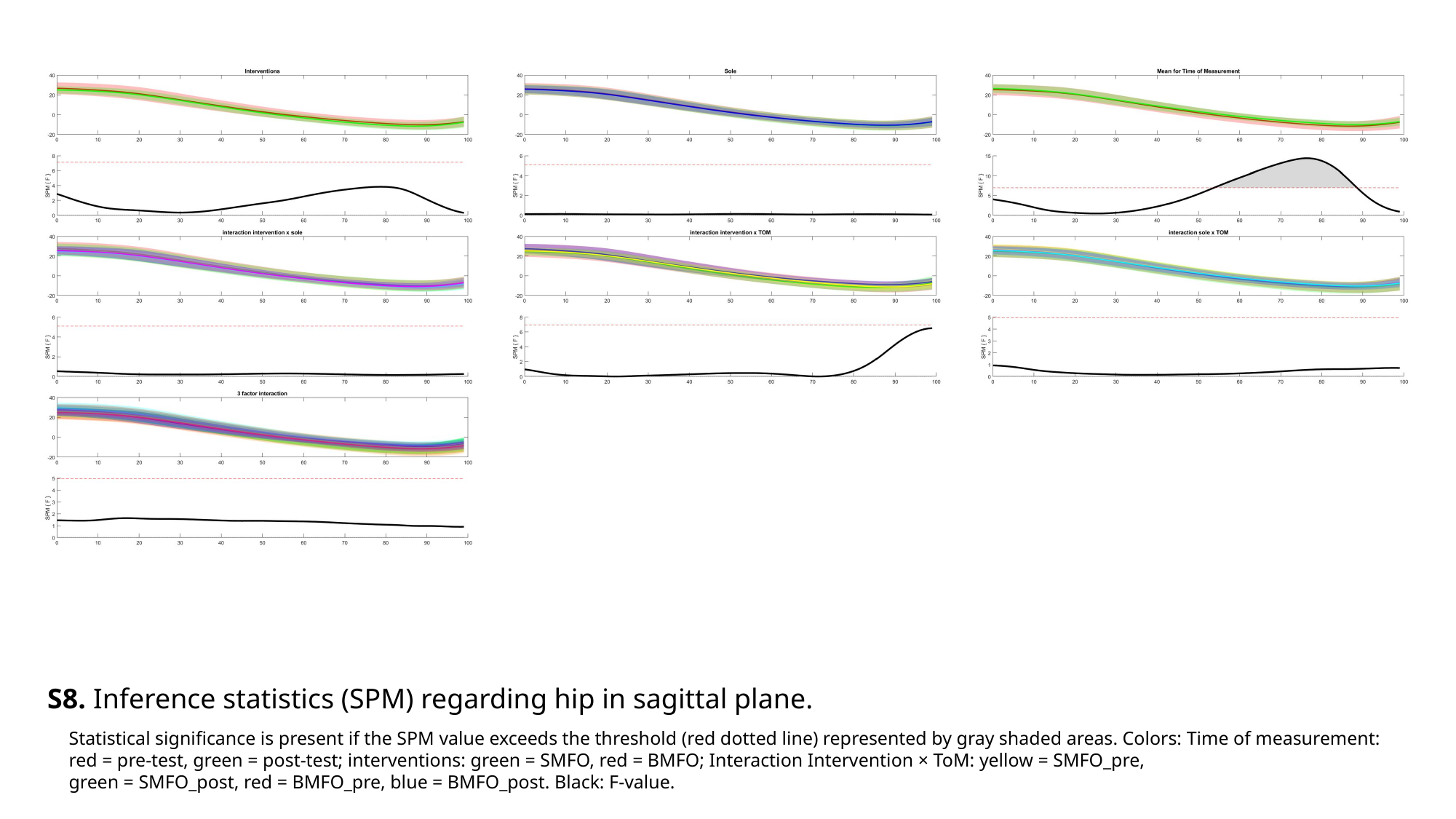

S8. Inference statistics (SPM) regarding hip in sagittal plane.
Statistical significance is present if the SPM value exceeds the threshold (red dotted line) represented by gray shaded areas. Colors: Time of measurement:
red = pre-test, green = post-test; interventions: green = SMFO, red = BMFO; Interaction Intervention × ToM: yellow = SMFO_pre,
green = SMFO_post, red = BMFO_pre, blue = BMFO_post. Black: F-value.

## Slide 9
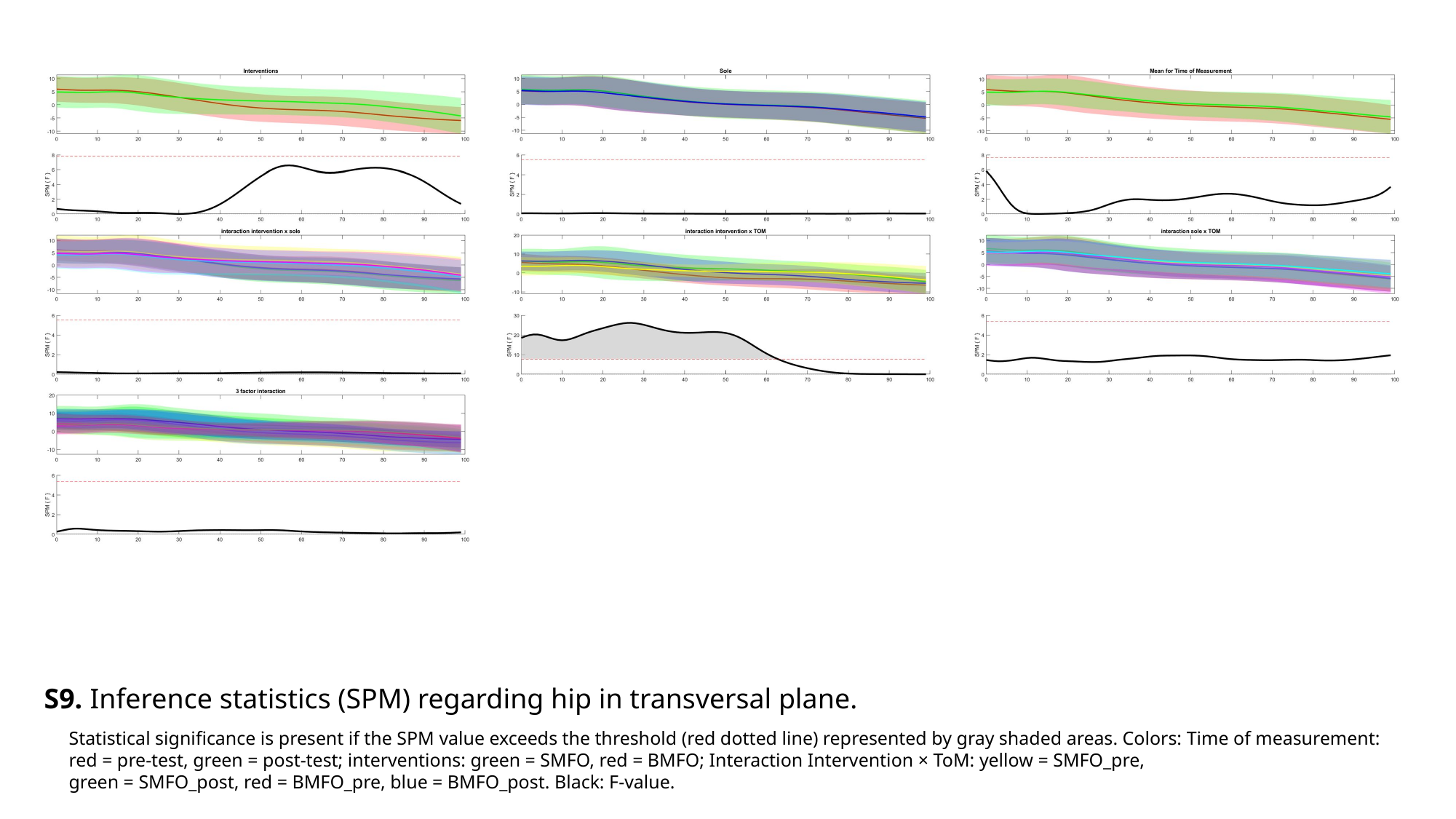

S9. Inference statistics (SPM) regarding hip in transversal plane.
Statistical significance is present if the SPM value exceeds the threshold (red dotted line) represented by gray shaded areas. Colors: Time of measurement:
red = pre-test, green = post-test; interventions: green = SMFO, red = BMFO; Interaction Intervention × ToM: yellow = SMFO_pre,
green = SMFO_post, red = BMFO_pre, blue = BMFO_post. Black: F-value.
